# The next steps in academic integrity — education, awareness, norms, duty and law

**DOI:** 10.1080/20961790.2021.1970887

**Published:** 2021-09-08

**Authors:** Yuehong (Helen) Zhang, Hanfeng Lin, Xinxin Zhang, Qing Ye

**Affiliations:** Journal of Zhejiang University-Science, Hangzhou, China

**Keywords:** Academic integrity awareness index, internet survey, COPE, journal, top university

## Abstract

We conducted and analyzed several internet surveys in order to understand the profile of global research integrity and ethical awareness, encompassing global population distribution. These were (1) the global distribution of Committee of Publishing Ethics (COPE) membership; (2) the global distribution of “Integrity” or “Ethics” journals; (3) the level of academic integrity awareness in European higher education institutions and (4) awareness of academic integrity in the top universities of Asia and Africa. The results of this survey series highlight seriously imbalanced awareness of research integrity and publishing ethics across the world, especially in developing areas with the highest population density. We therefore propose a new index, the “Academic Integrity Awareness Index” for future discussions across the linked spheres of publishing and research.

## Introduction

To nurture the ecological environment of academic integrity on a global level, regardless of the discipline, we firmly believe that strengthening education, raising awareness, establishing norms, highlighting duty and improving moral law are particularly nece­ssary. Along these lines, we conducted several surveys using internet search data to measure and analyse the status quo of global academic integrity consciousness (some data presented here were presented at the 6th World Conference on Research Integrity in 2019 (6th WCRI)). As of August 4, 2021, using the original survey methodology, the following internet survey information has been updated and supplemented. Specifically, we present the following: (1) global distribution of Committee of Publishing Ethics (COPE) membership on six continents; (2) global distribution of “Integrity” or “Ethics” journals; (3) academic integrity awareness in European higher education institutions and (4) academic integrity awareness in the top universities of Asia and Africa. Based on the present results and interpretation of these surveys, we are deeply aware that the global awareness of academic integrity is imbalanced, especially in densely populated areas in the world, all of which shows that research integrity will always coexist with scientific exploration and is always on the way. And more interesting and important in the survey, a new research integrity index seems to persist in our minds.

## Global distribution of COPE membership

Since the COPE’s inception in 1997, it has become a highly esteemed international organization that supports editors, publishers, and those involved in academic publishing ethics. COPE offers resources for its members from all academic fields. Therefore, our analytical approach was to first look at the distribution of the world’s population across six continents, and then measure COPE’s membership distribution in parallel, with the aim of analyzing level of awareness of ethical and integrity issues in the global scientific and technological publishing industry.

**Figure 1.**
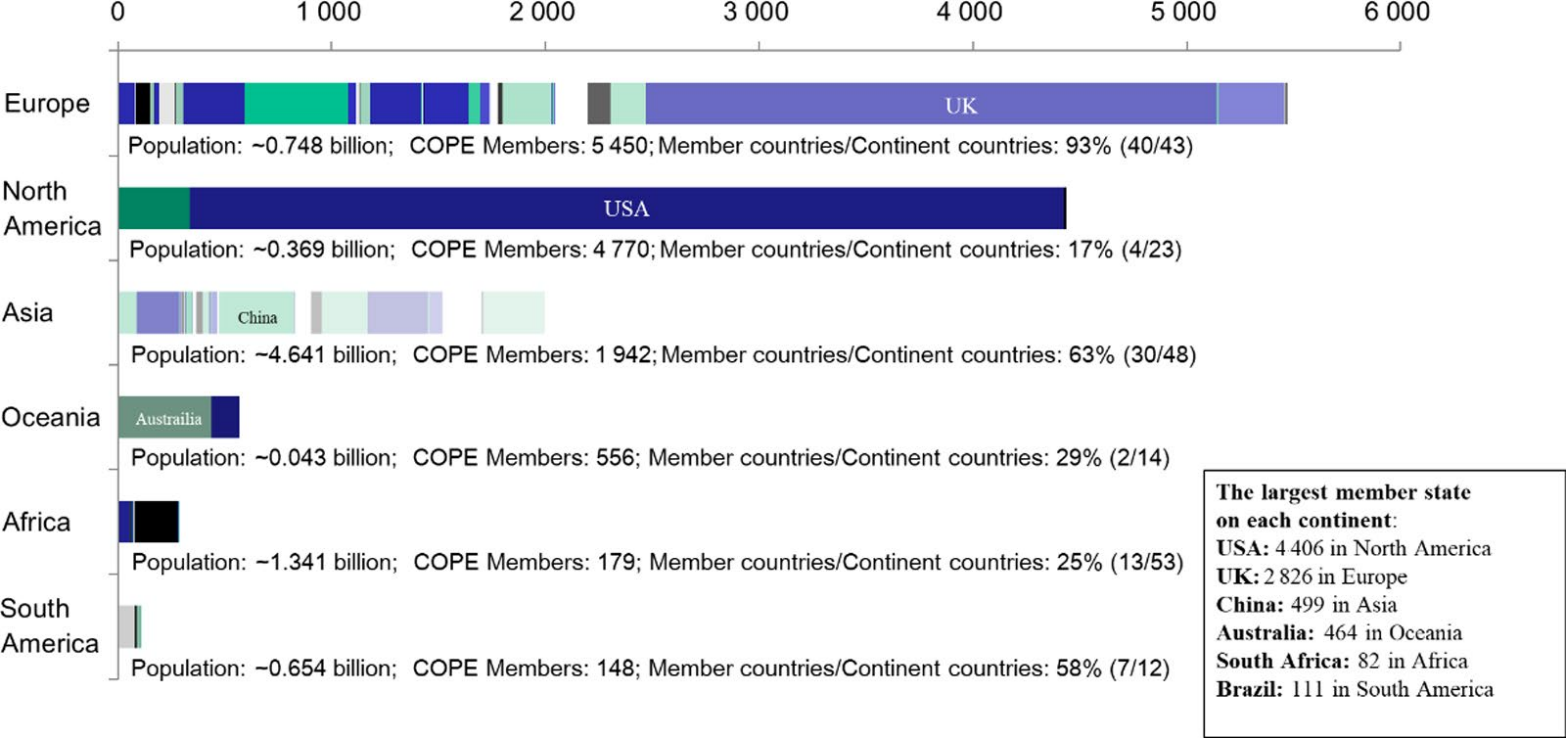
Distribution of Committee of Publishing Ethics (COPE) membership countries on six continents in 2021 (updated June 29, 2021). Note: Europe: population: ∼0.748 billion; COPE members: 5 450; member countries/continent countries: 93% (40/43); in which, the largest number of members is the UK 2 826, followed by Germany 541 and then France 299. North America: population: ∼0.369 billion; COPE members: 4 770; member countries/continent countries: 17% (4/23); in which, the largest number of members is the USA 4 406, followed by Canada 350 and then Mexico 11. Asia: population: ∼4.641 billion; COPE members: 1 942; member countries/continent countries: 63% (30/48); in which, the largest number of members is China 499 (including Hong Kong: 58; Taiwan: 49), followed by Iran 306 and then India 275. Oceania: population: ∼43 million; COPE members: 556; member countries/continent countries: 29% (2/14); in which, the largest number of members is Australia 464, followed by New Zealand 92. Africa: population: ∼1.341 billion; COPE members: 179; member countries/continent countries: 25% (13/53); in which, the largest number of members is South Africa 82, followed by Egypt 67 and then Nigeria 7. South America: population: ∼0.654 billion; COPE members: 148; member countries/continent countries: 58% (7/12); in which, the largest number of members is Brazil 111, followed by Columbia 20 and then Argentina 7. The largest COPE member state on each continent: USA: 4 406 in North America; UK: 2 826 in Europe; China: 499 in Asia (including Hong Kong and Taiwan); Australia: 464 in Oceania; South Africa: 82 in Africa; Brazil: 111 in South America.

Current United Nations Population Data show that the world population has exceeded ∼7.79 billion, and that half of the population under the age of 30 live in developing countries [1, 2]. From the perspective of world population density, we looked at the distribution of COPE members on six continents. As of June 29, 2021, the total registered membership of COPE had reached 13 065 worldwide (institutions and individuals). According to our statistics, the ratio of COPE membership to the popu­lations in the six continents is distributed as follows: 5 450 members/748 million in Europe, 4 770/369 million in North America, 1 942/4 641 million in Asia, 556/43 million in Oceania, 179/1 341 million in Africa and 148/654 million in South America (Figure 1).

As shown in the note in Figure 1, clearly, COPE membership is out of proportion to population density. The highest COPE membership occurs in Europe (5 450) and North America (4 770), with the two primary countries in each place being the UK (2 826) and the USA (4 406). If we take COPE membership as an index of Academic Integrity Awareness in publishing industry and compare the most densely populated continents Asia and Africa, even though China has the highest membership in Asia (499), and South Africa has the highest on the African continent with 82, these indexes are not enough. Whether it is reasonable for us to propose the Academic Integrity Awareness Index as a first step will be discussed in this paper.

## Global distribution of “Integrity” or “Ethics” journals

**Figure 2.**
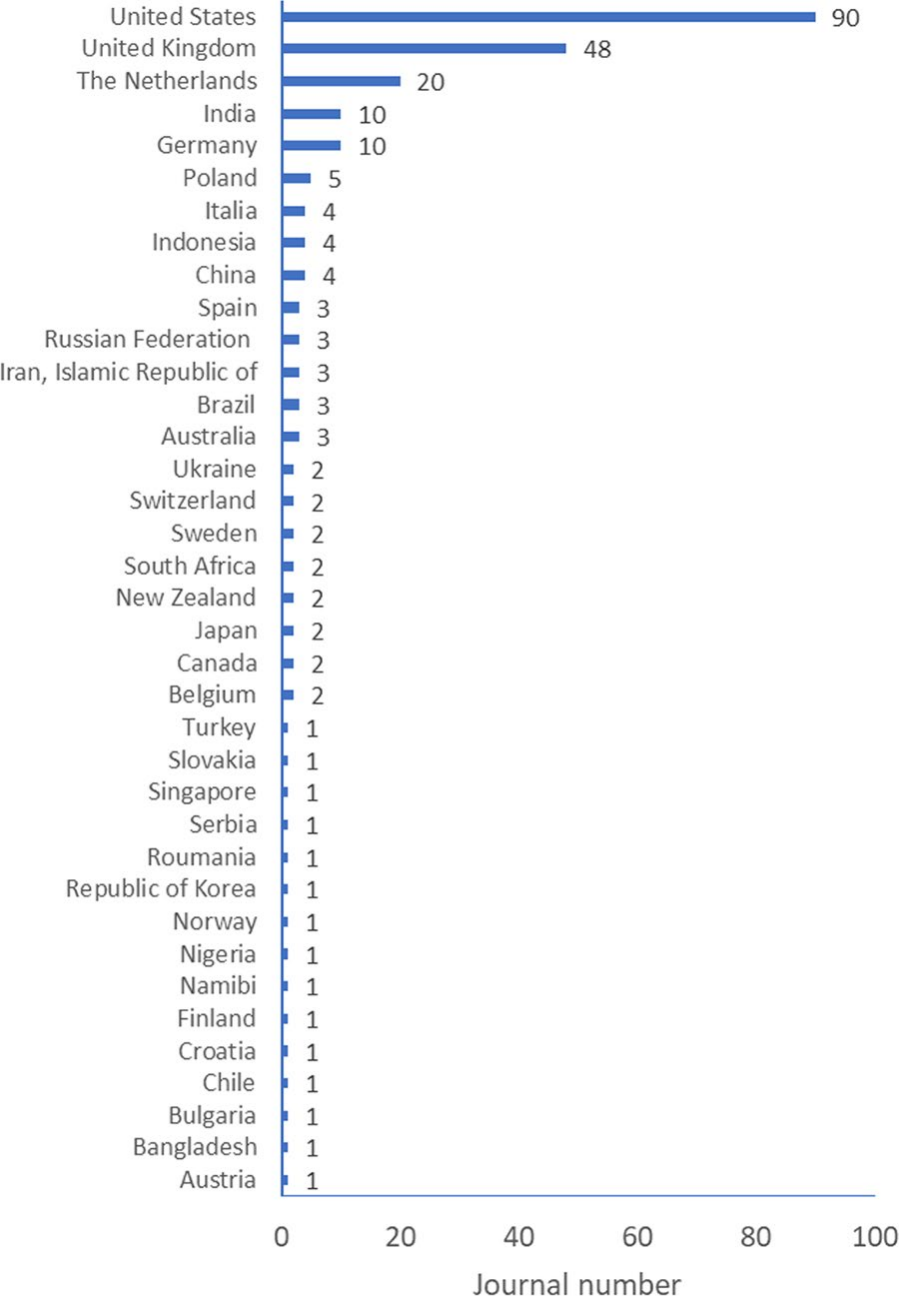
Distribution of journals with “ethics”, “moral” and “integrity” in titles based on searching *via* academic category of Ulrich’s Periodicals Directory Database (http://ulrichsweb.serialssolutions.com, updated on August 4, 2021). The summed journal number is 241 rather than 237 because some journals have two International Standard Serial Numbers (ISSNs), one for print version and the other for online version, which are registered in different countries.

For the Academic Integrity Awareness Index proposed in this paper, our second step was to elucidate the number of academic journals on this topic that are distributed around the world. In 2019, we did a survey to find 114 academic journals by searching with two key words, namely, “ethics” and “integrity” on Scopus and JCR databases, which had been posted at the 6th WCRI (https://wcrif.org/wcri2019) and published in Chinese [3]. For this paper, in addition to “ethics” and “integrity”, we added the third key word “moral”, and expanded our search using the same methodology to include the world’s largest database, Ulrich’s Periodicals Directory Database (http://ulrichsweb.serialssolutions.com). By manual comparison of these journals’ publication scope with key words “ethics”, “integrity” and “moral”, there are in total 237 active journals in Ulrich’s academic category, which has about 162 794 journal titles. Figure 2 shows 237 journals, most of them in English, of which the USA has the most journals (90), followed by the UK (48), The Netherlands (20), India (10) and Germany (10), with less periodicals from developing countries. These data correlate to data shown in Figure 1 on COPE membership distribution. As shown in Figure 1, the number of journals related to research integrity and ethics topics in the world’s most densely populated areas is insufficient. We believe this presents signifi­cant evidence of the research integrity awareness index in the world.

## The level of academic integrity awareness in European higher education institutions

**Figure 3.**
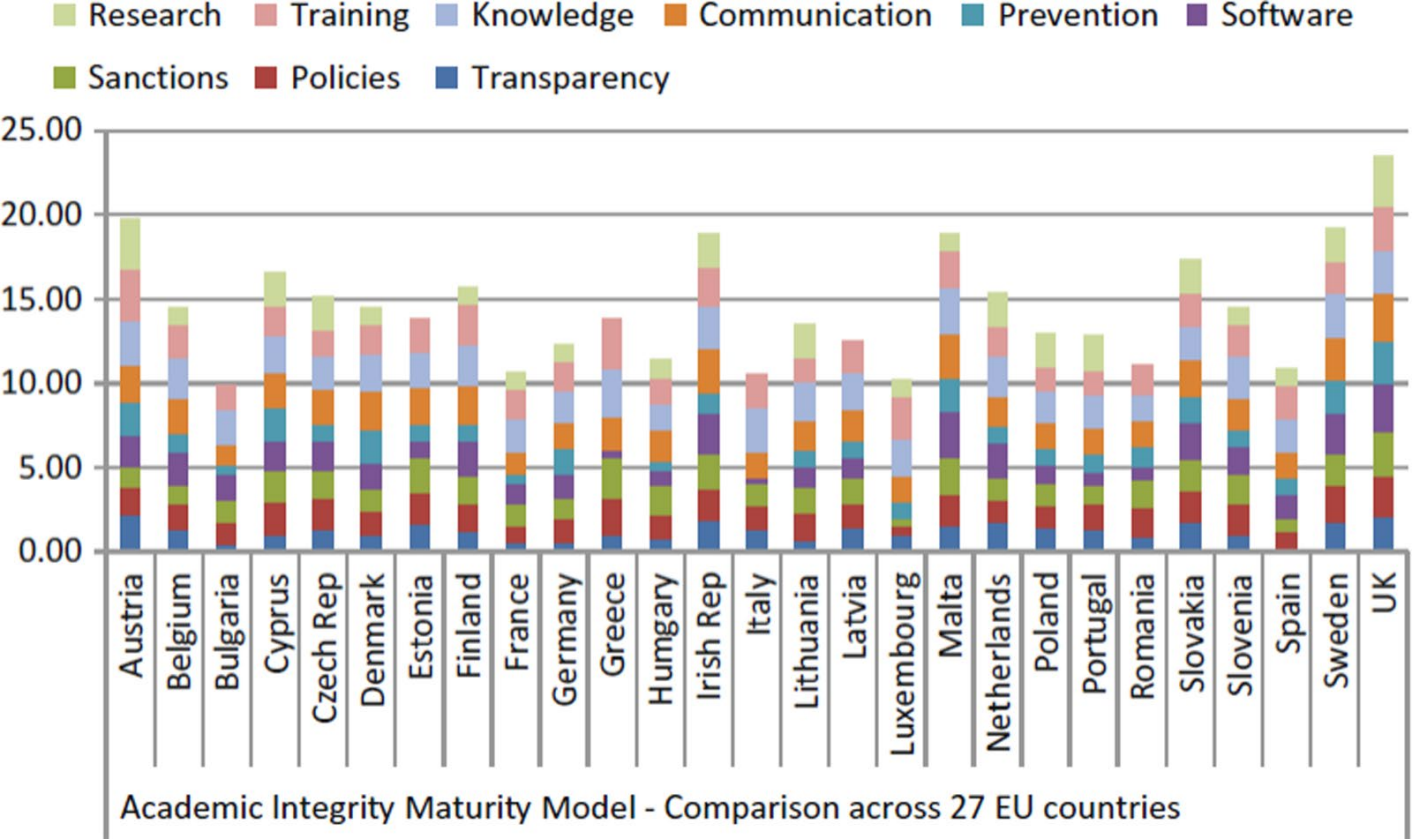
Impact of Policies for Plagiarism in Higher Education Across Europe (IPPHEAE) Project, Academic Integrity Maturity Model (AIMM) 27 County Comparison [4] (Reproduced from Springer with permission in 2019).

As we discuss the challenging topic of academic integrity in the global ecological research environment, we must consider the relationship between education, awareness, and academic norms. First, we know that education can raise awareness of research integrity, but education is seriously imba­lanced globally. Let us understand first how academic integrity is perceived and managed in European higher education institutions. Here, we can refer to the European (27 countries) Perspectives of Academic Integrity Maturity Model (AIMM) that was based on the project entitled, “Impact of Policies for Plagiarism in Higher Education Across Europe” (IPPHEAE 2010–2013), which aimed to explore how academic integrity was understood and managed in different parts of the European Union (EU) [4]. The comparison of results from the different countries was achieved using a specially designed toolset called AIMM where results for each country were tabulated and charted using a stacked bar chart for the 27-country comparisons. This enabled depiction of the results for each of the nine categories related to research integrity awareness topics in each country, as shown in Figure 3 [3].

These data elucidate the fact that across 27 EU countries, as far as education and raising awareness of research integrity based on the nine aspects (research, training, knowledge, communication, prevention, software, sanctions, policies and transpa­rency), the level of consciousness varies greatly. Although there have been significant advances and innovation in research and policy improvements on research integrity in Europe in recent years [5, 6], controversy still exists [7], and an imbalanced awareness of research integrity across the 27 EU countries still persists.

## Awareness of academic integrity in the top universities of Asia and Africa

Building upon results from the previous surveys, our next interest was to understand how the top universities in Asia and Africa, two continents with large population densities and many young people, are aware of research integrity. In short, our original intention was to observe whether there are transparent research integrity guidelines and policies in these top universities’ education systems. First, according to data presented on current websites of Statistics Times 2020 GDP Ranking (http://statisticstimes.com/economy) and 2021 Times Higher Education (THE) (https//www.timeshighereduction.com), we selected the top 20 countries in Asia and Africa, respectively, and then selected the top 10 universities in each country. If less than 10 or none occurred in THE, we referred to the other resource (UniRank) to reflect the local research integrity and ethics education information. Using the methodology described above, we followed the approach of searching the internet for each university to complete the two lists below, expanding information from Asia and Africa.

### Top universities research integrity information via their websites in Asia

In our internet survey of Asia, we gathered these data from the following two ranking lists as the survey’s references:

1. Top 20 countries and two regions (Taiwan and Hong Kong, China) from the List of Asian countries by 2019 nominal GDP (International Monetary Fund World Economic Outlook (October 2019), http://statisticstimes.com/economy/asian-countries-by-gdp.php, accessed on August 4, 2021).
2. We selected top 10 universities of each country/region from the THE Asia University Rankings 2021, but here some countries have less than 10 universities on the list (https://www.timeshighereducation.com/world-university-rankings/2021/regional-ranking#!/page/0/length/25/sort_by/rank/sort_order/asc/cols/stats, accessed on August 4, 2021).

Browsing the above-referenced websites, we conducted a cursory review of the policies and regulations related to academic integrity or ethics on the official university website, and then searched the key words “integrity”, “ethics”, “plagiarism” or “misconduct” from the official university website *via* Google or Baidu or Bing. It is worth noting that the search results of “not found” does not mean that the university website does not provide information, but simply that it was not found using those search terms.

In sum, we retrieved documents offering research integrity information on the official websites of 75% (108/144) of the top universities in the top 20 Asian countries (including Taiwan and Hong Kong regions, China) by 2019 Nominal GDP, which implies that most Asian universities attach great importance to research integrity education (Table 1).

**Table 1.** Research integrity documents retrieved from top university websites in Asia.

GDP rank	Countries/regions	All universities in THE list	Top universities included	Academic integrity information	
				Found	Not found
1	Chinese mainland	91	10	10	0
2	Japan	116	10	10	0
3	India	63	10	10	0
4	The Republic of Korea	35	10	9	1
5	Indonesia	9	9	3	6
6	Saudi Arabia	10	10	6	4
7	Chinese Taiwan	38	10	10	0
8	Thailand	17	10	6	4
9	Islamic Republic of Iran	47	10	3	7
10	United Arab Emirates	5	5	5	0
11	Israel	7	7	5	2
12	Chinese Hong Kong	6	6	6	0
13	Malaysia	15	10	9	1
14	Singapore	2	2	2	0
15	The Philippines	2	2	2	0
16	Bangladesh	2	2	0	2
17	Pakistan	16	10	6	4
18	Vietnam	3	3	1	2
19	Iraq	3	3	1	2
20	Qatar	1	1	1	0
21	Kazakhstan	3	3	2	1
22	Kuwait	1	1	1	0
Total		492	144	108 (75%)	36

THE: Times Higher Education.

### Top universities research integrity information via their websites in Africa

In the internet survey of Africa, we used the similar approach as with Asia, and only noted the references listed below:

1. Top 20 countries from the List of African countries by nominal GDP rankings are from the International Monetary Fund World Economic Outlook (October 2019). (http://statisticstimes.com/economy/african-countries-by-gdp.php, accessed on August 4, 2021).
2. We selected top universities from the THE’s Best Universities in Africa 2021 (https://www.timeshighereducation.com/student/best-universities/best-universities-africa, accessed on August 4, 2021). The best universities of Nigeria, South Africa, Egypt, Algeria, Morocco, Kenya, Ghana, Tunisia and Uganda were listed only from the THE. The other countries’ top universities were selected from the UniRank (https://www.4icu.org/Africa, accessed on August 6, 2021), according to their GDP rankings in Africa.

We found research integrity documents on the official websites of only 33.7% (32/95) top universities from the top 20 African countries by 2019 nominal GDP, which implies that most of the universities in Africa should pay more attention to research integrity education (Table 2).

**Table 2.** Research integrity documents retrieved from top university websites in Africa.

GDP rank	Countries	Top universities investigated	Academic integrity information	
			Found	Not found
1	Nigeria	12	5	7
2	South Africa	13	13	0
3	Egypt	15	2	13
4	Algeria	16	4	12
5	Morocco	10	1	9
6	Kenya	3	2	1
7	Angola	2	1	1
8	Ethiopia	1	1	0
9	Ghana	1	1	0
10	Tanzania	1	0	1
11	Democratic Republic of the Congo	1	0	1
12	Côte d’Ivoire	2	0	2
13	Tunisia	6	0	6
14	Cameroon	2	0	2
15	Libya	2	0	2
16	Sudan	3	0	3
17	Uganda	1	0	1
18	Zambia	1	1	0
19	Senegal	2	0	2
20	Botswana	1	1	0
Total		95	32 (33.7%)	63

Similar to Asia, we searched key words “integrity”, “ethics” and “plagiarism” on the official university website using Google or Baidu. For some, where university websites only used French and Arabic, we searched key words translated into French, “intégrité”, “éthique” or “plagiat”, corresponding to integrity, ethics and plagiarism in English. Again, the fact that search results indicated “not found” does not meana lack of information, but merely that no information was found using those terms. Moreover, we only counted the universities that have polices/handbook/manual on integrity/ethics/plagiarism. We did not count the universities that launched conferences, workshops or publishing centre or those that use anti-plagiarism tools (e.g. iThenticate, Turnitin), or introduced academic integrity books to students, such as Suez Canal University (Egypt), Ain Shams University (Egypt) and University of Rabat (Morocco). Nevertheless, we were pleased to observe that as reflected by their websites, many universities in Africa are starting to take academic and research integrity seriously.

From the above-mentioned data, we can be certain that across the six continents, there is a serious imbalance in education and awareness on publishing ethics and research integrity. Although, Asia and Africa are the two continents with the highest popu­lation density and contain many developing countries, we find the hope in the fact that both continents are making progress in this effort in the past few years, especially in Asia where the percen­tage of retrieved research integrity documents from the top university websites is 75%, as compared to 33.7% in Africa, suggesting more work needs to be done to raise awareness, especially in Africa.

## What are the next steps?

We know that education, awareness and norms of research integrity are critically important. Building upon these important values, what should our next steps be as we proceed in support of academic integrity? In this paper, we have presented a new index, the “Academic Integrity Awareness Index” for discussion as one of many potential next steps on behalf of the global community committed to integrity. Although we believe this will contribute to fostering an environment of integrity, we recognize that it is not enough.

Since the 17th century gave rise to normative behaviour among scientists [8], scientific research integrity has become a significant topic of public interest. The issue is still discussed at the moral level today, however, sometimes as if no law is involved [9]. This quandary is similar to copyright issues, recognizing that economic rights are featured in the foreground in legal measures, while moral rights may be less prominent. For example, the Trade Related Aspects on Intellectual Property Rights (1994) contains no provisions on moral rights within its articles [10]. Thus, in exploring next steps, as human society advances with The Times from internet technology to ideology, let us consider how the whole culture of Academic Integrity Awareness from education, awareness, norms to duty and law might be improved. Of course, this is a complex research topic to be discussed among academic organizations and experts in each field, including those in diverse fields of science and law.

## Authors’ contributions

Yuehong (Helen) Zhang contributed to the project planning and supervision, and wrote and reviewed the whole paper. Hanfeng Lin wrote the top universities research integrity information of Asia. Xinxin Zhang wrote the top universities research integrity information of Africa. Qing Ye contributed to the data collation and diagramming of COPE membership and ethics journals.
